# Editorial: Advances in the research of diabetic nephropathy

**DOI:** 10.3389/fendo.2022.1116188

**Published:** 2023-01-05

**Authors:** Mohamed Abu-Farha, Katsumi Iizuka, Daisuke Yabe, Fahd Al-Mulla, Jehad Abubaker

**Affiliations:** ^1^ Biochemistry and Molecular Biology Department, Dasman Diabetes Institute, Dasman, Kuwait; ^2^ Department of Clinical Nutrition, Graduate School of Medicine, Fujita Health University, Toyoake, Japan; ^3^ Department of Diabetes and Endocrinology, Gifu University Graduate School of Medicine, Gifu, Japan; ^4^ Dasman Diabetes Institute, Dasman, Kuwait

**Keywords:** diabetic nephropathy, lipidomic, cystatin C (Cys C), N-cadherin expression, EGFR

The increased prevalence of diabetes is associated with an increased incidence of diabetic nephropathy, which is estimated to affect approximately 40% of patients with diabetes. Diabetic Nephropathy, or Diabetic Kidney Disease (DKD), refers to the deterioration of kidney function in patients affected by type 1 and type 2 diabetes. To date, diabetic kidney disease is the leading cause of kidney failure and the single highest cause of diabetic mortality. Nevertheless, research has yet to reveal a definitive mechanism for the association between hyperglycemia and damage to the kidneys.

Through this Research Topic, the heterogeneity of diabetic nephropathy etiology and the underlying molecular mechanisms was explored. To this end, genetic and epigenetic factors associated with nephropathy were considered, as well as the role of oxidative stress and ferroptosis. Also pertinent to this topic was how the oxidative-stress pathway can be modulated to prevent or reverse diabetic nephropathy. Additionally, a special focus was given to early biomarkers that can lead to a better understanding and early detection of the disease.

In this special issue, several articles focused on the use of early diagnostic markers for diabetic nephropathy. To use lipidomics to compare the kidney cortex of normal and diabetic rats, Hou et al. showed a unique signature of lipid molecules associated with DKD kidney. They used targeted lipidomic approach spanning 437 lipid species and 25 lipid classes to study changes in the kidney cortex in normal and DKD rat model. The main characteristics of DKD lipidome are changes in side chain composition and unsaturated bonds. Neutral lipids exhibiting a higher degree of unsaturation and side chains of linoleic acid were the most lipids associated with DKD. Additionally, Glyceride lipids, lysophospholipids, and sphingolipids showed a significant increase in the DKD kidney cortex. Taken together, the authors documented lipid changes associated with increased kidney damage, which can be used to better understand the pathophysiology and early detection of kidney damage in people with diabetes.

In another study, Wang et al. explored, in a retrospective cohort, the association of the trajectory of serum Cystatin C level with diabetic kidney disease development [2]. It was shown that the Cystatin C level was higher in people with diabetes. Additionally, once the people with diabetes were divided into low, middle, and high increasing Cystatin C levels, people in the middle and high increasing Cystatin C classes had higher incidence of diabetic kidney disease.

Furthermore, Wei et al. and Ali et al. showed that increased serum VEGF-B level and N-Cadherin levels were associated with diabetic kidney disease [3; 4]. Wei et al. showed that the circulating level of VEGF-B was associated with renal impairment. The diabetic population was divided based on eGFR, showing that serum VEGF-B level was an independent risk factor of eGFR<90 mL/min/1.73m2. Ali et al. measured the plasma level of N-Cadherin in a group of healthy controls and in people with T2D with and without DKD. It was shown that the plasma level of N-Cadherin was significantly higher in the DKD compared to the diabetic patients without DKD, and with the non-diabetic control group [4].

Additionally, a few articles also focused on the pathophysiology of DKD. Zhang et al. utilized RNA-Seq to identify novel genes involved in developing diabetic kidney diseases. They measured mRNA gene expression from glomeruli isolated from db/db and db/m mice with albuminuria. Gene expression analysis showed that genes upstream of glycolysis, such as *Hk1* and *Pfkp*, were upregulated, while genes downstream of glycolysis, such as Pkm and *Ldhaw*, were downregulated [5]. *PFKP* was shown to play a protective role against podocytes damage through the production of fructose-1,6-bisphosphate. Exogenous fructose-1 and 6-bisphosphate administration was also associated with improved kidney injury caused by high glucose cytoskeletal remodeling in podocytes [5]. Taken together, the authors demonstrated the potential for targeting *PFKP* for the treatment of DKD.

To elucidate the role of long non-coding RNA in DKD, Yang et al. focused on a previously identified lncRNA that high-throughput RNA-seq identified. The authors showed that the RNA component of mitochondrial RNAase P (Rmrp) lncRNA was highly expressed in the kidneys of db/db DKD mice and glomerular mesangial cells. They showed that Rmrp was controlled at the transcriptional level by transcription factor Sp-1. Rmrp up-regulated JunD expression through sponge miR-1a-3p, which may contribute to mesangial cell proliferation and fibrosis in DKD.

Increased reactive oxygen species and lipid peroxidation has been linked to an iron-dependant cell death called ferroptosis. Feng et al. linked ferroptosis and renal damage in diabetic mice to an increased HIF-1α/HO-1 pathway activity. Two studies investigated the role of autophagy in the development of kidney damage. Tang et al. investigated the role of melatonin in the clearance of damaged mitochondria in the kidney. The authors first showed that diabetic mice with DKD had abnormal mitophagy accompanied by increased oxidative stress and inflammation. Melatonin alleviated kidney damage by promoting AMPK phosphorylation and the translocation of mitophagy associated proteins PINK1 and Parkin to the mitochondria [8]. Wang et al. investigated the impact of hyperglycemia during a 6 h hyperglycemic clamp where blood glucose was increased from normal blood glucose of 5.37 ± 0.52 mmol/L to 11.67 ± 1.21 mmol/L, 16.67 ± 2.11 mmol/L, 24.73 ± 3.43 mmol/L. The authors showed that acute hyperglycemia resulted in renal tubular injury *via* mitophagy AMPK/mTOR pathway inhibition. They also showed that this damage could be inhibited by AMPK activation or mTOR inhibition.

## Author contributions

MA-F drafted the editorial. All authors contributed to editing the manuscript. All authors contributed to the article and approved the submitted version.

